# Impact of COIL: Learning From Student Nurses in Norway Who Collaborated With U.S. Students

**DOI:** 10.1177/10436596231209043

**Published:** 2023-11-07

**Authors:** Unni Jenssen, Jeanie M. Bochenek, Tara Spalla King, Simen A. Steindal, Ida Voss Hestvold, Dianne Morrison-Beedy

**Affiliations:** 1 Lovisenberg Diaconal University College, Oslo, Norway; 2 The Ohio State University College of Nursing, Columbus, USA; 3 University of South-Eastern Norway, Drammen, Norway

**Keywords:** internationalization, global nursing education, collaborative online international learning (COIL), qualitative research, cultural awareness, virtual global classrooms

## Abstract

**Aim:**

The aim of this study was to describe the experience of Norwegian nursing students with internationalization through participation in a Collaborative Online International Learning (COIL) course.

**Background:**

Educators in Norway and the United States collaborated to incorporate internationalization and population health concepts into virtual courses during the pandemic. Literature gaps exist in post-implementation assessment data that ascertain internationalization through the COIL experience.

**Design:**

This was a qualitative study with a descriptive design. Data were collected from focus group interviews and analyzed conventional content-analysis approaches.

**Methods:**

Fifteen Norwegian undergraduate nursing students who participated in the COIL opportunity completed focus group interviews.

**Findings:**

The themes identified included, “virtual conversation builds collaborations and enhances learning,” and “this opened my eyes.”

**Conclusions:**

Norwegian students acknowledged they had learned transferable lessons from their global partners that could be applied to patient care of the marginalized population in Norway.

## Background

Global learning for nursing students in higher education is imperative, as nurses need knowledge and skills that address the complex needs of diverse populations (Dorri et al., 2020). Nurse educators in Norway and the United States recognize the importance of incorporating internationalization and population health concepts into nursing curriculum to effectively manage global health needs and reduce associated health inequities (Morrison-Beedy et al., 2020).

Prior to the COVID-19 pandemic, approximately 10% of U.S. students studied abroad (Open Doors, 2018); however, less than 1% studied abroad during the 2020-2021 academic year (Open Doors, 2022). Similarly, in Norway, the pandemic had severe ramifications for internationalization in higher education and about 55% of international activities came to a halt, while around 33% continued with different learning methods (DIKU, 2021). Due to the COVID-19 pandemic, institutions had to be creative and use alternative learning methods for nursing students to achieve their internationalization learning goals (Wallace et al., 2021).

### Collaborative Online International Learning

The American Council of Education (ACE) and the Norwegian Agency for International Cooperation and Quality Enhancement in Higher Education (DIKU) developed and offered an intensive 3-month online Transformation Lab to provide U.S. and Norwegian partner Institutions the skills and resources to learn and apply quality-assured Collaborative Online International Learning (COIL) exchange program. COIL refers to an approach to internationalization provided by two or more international higher institutions offering virtual collaboration (Gray et al., 2021; Misra et al., 2020). It is a teaching and learning modality that brings students and faculty together despite various cultures, time zones, language barriers, and international borders, with the intention that students can learn, discuss, and collaborate with each other (Onoverole & Fowler, 2021). Learning, understanding, and developing global competence take center stage while institutions choose specific virtual tools.

### Cultural Competence

Knowledge of cultural competence theory is vital to frame COIL. Campinha-Bacote’s (2018) cultural theory highlights the synergy between cultural humility, an inward reflection coupled with personal assessment, and cultural competence in a dynamic and interactive process. Key terms include cultural encounters, awareness, skill, and desire. Cultural encounters are face-to-face interactions with other cultures that may revise our beliefs, forming the crux of our cultural competence journey. Cultural awareness is looking at our biases and backgrounds and being aware of racism. It occurs when we build an educational base of information about diverse cultural groups. Cultural skill is sensitively collecting physical, spiritual, and psychological data about culturally diverse others. Cultural desire occurs within and provides excitement and motivation to continue to engage with and learn about other cultures (Campinha-Bacote, 2020).

### Our Norway/U.S. COIL Collaboration

To continue offering our students internationalization during COVID-19, faculty and instructional designers, who experienced the immersive Transformation Lab utilizing best practices in online education, developed a COIL course together. The COIL was suitable for our U.S. and Norwegian students on urban health, focusing on the marginalization of people living in cities. Marginalized populations refer to groups who are socially and economically excluded from the society in which they live (Jenson, 2000). The U.S. and Norwegian COIL teams created a common syllabus, learning objectives, and learning activities that met mutual curriculum requirements (Table 1). The COIL course provides students with key cultural informants, videos about resources in the community to support marginalized populations, and other educational material to be used as they progress through the course and complete a common project.

**Table 1:** Crosswalk Between Course Learning Objectives, Activities, Content, Assignments, Associated Interview Questions, and Theoretical Base.

COIL Urban Health Course Description			
Through the course students will work together on a project to: - Explore Sustainable Development Goals (SDGs), specifically SDG #3: Ensure healthy lives and promote well-being for all at all ages. - Gain insight into living marginalized. - Self-reflect on values as a nurse regarding marginalized groups. - Discuss, compare, and contrast differences and similarities between cultures. - Learn about healthcare and nursing practices between cultures.			
Ice breakers	Type of Activity	Content & Assignments	Associated Interview Questions
- Icebreaker 1 (cultural encounter, cultural awareness & cultural desire Campinha-Bacote, 2020)	- Synchronous Meet and greet: - The meeting will be recorded.	- Inform about COIL - Presentation of students and faculty - Groups of 4 meet in breakout rooms - Getting to know each other activities	- What are your thoughts about the icebreaker activities? - How might those icebreaker activities have impacted or not your working relationships or team building with your international peers? - Any other thoughts about connecting with students from LDUC/OSU? (Cultural encounter & cultural desire, Campinha-Bacote, 2020)
- Icebreaker 2 (cultural encounter, cultural awareness & cultural desire, Campinha-Bacote, 2020)	- Asynchronous - Introduction and student life	- Create individual 3- to 5-min short video, post on Canvas. - Introduce yourself and e.g., where you are from, your university, family life, friends.	
- Icebreaker 3 (cultural encounter, cultural awareness & cultural desire, Campinha-Bacote, 2020)	- Asynchronous - Introduction to a holiday	- Create a 3- to 5-min short video with a peer-student, post on Canvas. - Introduce a holiday	
- Icebreaker 4 (cultural encounter, cultural awareness & cultural desire, Campinha-Bacote, 2020)	- Synchronous - Case study, homelessness	- Groups of four discussing comparing, and contrasting the case study	
Learning Outcomes	Other Competencies	Content	Associated Interview Questions
After completing the course, the student should be able to: - explain social and socio-economic global challenges and inequality in health in big cities and consequences of these and adapt global health challenges (cultural knowledge, Campinha-Bacote, 2020) - assess health, education, work and living conditions in the planning and practice of nursing care for individuals and groups in society in a culturally competent perspective (cultural skill, Campinha-Bacote, 2020) - assess health-promoting, preventive and work-inclusive measures for marginalized citizens in the light of basic nursing and in a culturally competent perspective (cultural skill, Campinha-Bacote, 2020)	The students will: - exchange views, knowledge and experiences with fellow international students and teachers (cultural awareness, cultural knowledge, Campinha-Bacote, 2020) - formulate key subject matter in writing or orally through relevant forms of expression - discuss current challenges for the health and welfare services related to marginalization (cultural knowledge, Campinha-Bacote, 2020) - develop evidence-based strategies to be implemented in health promotion, risk reduction, and disease prevention in the population-focused care of individuals, families, groups, communities, and populations (cultural skill, Campinha-Bacote, 2020) - collaboratively work to solve issues of identified problems within marginalized populations (cultural skill, Campinha-Bacote, 2020)	- Explore determinants of health and disenfranchised populations. - Understand how access to care and support is driven by policies that could be biased. - Identify strategies used for health promotion, risk reduction, and disease prevention across populations. - Examine legal and professional issues that arise in caring for populations. - Explore the economic, societal, and political influences on the health of populations. - Compare and contrast the topics above from a global perspective.	- What would you like to share about working collaboratively with your international peers to solve problems for marginalized populations? - What would you like to share about your exposure to views, knowledge and experience of fellow international students and teachers? - What would you like to share about your *understanding of other cultures* through your virtual global classroom? - What would you like to share about *communicating with other cultures* through your virtual global classroom? (Cultural encounters, cultural desire, cultural knowledge, cultural skill, cultural awareness, Campinha-Bacote, 2020)

### Student Recruitment

The target groups were undergraduate students scheduled for Lovisenberg Diaconal University College’s (LDUC)urban health course and graduate students in an accelerated nursing program at Ohio State University (OSU) in a similar course. Student recruitment was conducted via the college website, orally, and on the students’ learning management system. Students were informed they would have to work on their COIL project beyond scheduled course times and sometimes in the evenings. Students applied by submitting an essay expressing reasons for seeking the COIL experience.

### Structure and Content

The course started with a synchronous Zoom meeting where students were introduced to COIL and reviewed learning outcomes, course design, course expectations, joint project guidelines (Table 1), and a case study on homelessness (Institute for Health care Improvement, n.d.). The case study was used as a segue for students to discuss resources from each country that support marginalized groups. In 2021 and in 2022, eight students from each country participated.

We developed icebreaker activities to facilitate common ground and allow students to get to know each other before the COIL project (Table 1). The design of these activities increases student motivation for learning, helps students form relationships (Kavanagh et al., 2011; Pratama et al., 2021), and allows Norwegian students to speak English without having to think about academic vocabulary (Chowdhury, 2022; Kavanagh et al., 2011). The COIL course was delivered over 2 weeks with lectures and associated resources developed by faculty and organized by instructional designers from each country (Table 1).

Students were divided into small international groups of four per Yamagata-Lynch, (2014), as well-structured small groups to help students better connect with their peers and attend course activities. Each group selected a common theme faced by marginalized populations they wanted to investigate. Two faculty checkpoints occurred in the 2-week period, whereby each group shared their project, giving students feedback and assurance they were on track. At the last meeting, each student group presented their project, comparing the concerns, resources, interventions, and proposed solutions from each country related to the common theme selected. While the focus for students was to work on urban health and meet the COIL course’s common learning outcomes, an additional outcome was to develop a skill set and understanding that would allow them to work toward global competence in nursing.

### Study Purpose

While a few studies describe COIL projects in nursing (Davis et al., 2022; Kiegaldie et al., 2022), none entail collaborations between Norway and the United States. Furthermore, this work is unique in that it presents qualitative post-implementation assessment data. The purpose of this study was to describe Norwegian nursing students’ experiences with international learning and understanding through participation in this COIL course.

## Method

To gain insight into the students’ experiences, responses, and understanding of the COIL educational approach, we conducted a qualitative study with a descriptive design using focus group interviews with 15 Norwegian student participants. These carefully planned discussions, provided in private, safe, known spaces, are designed to understand the perspectives, challenges, and motivations in participation of the students who voluntarily selected to participate in a COIL opportunity within their required coursework (Cote-Arsenault & Morrison-Beedy, 1999). The social interaction among the participants in focus groups can facilitate associations that allow comparison of reflections among the group members, compared with individual interviews (Krueger & Casey, 2000). The Consolidated criteria for reporting qualitative research, a checklist for reporting studies using interviews and focus groups, guided the reporting of this study (Tong et al., 2007).

### Ethical Considerations

This study included only Norwegian students and was approved by the Norwegian Center for Research Data. The students were informed and invited to participate in a focus group interview when they agreed to participate in the COIL by signing a consent. Students were informed that the interviews were to learn more about the COIL experience as an international pedagogical learning method and how it could be revised for continuous quality improvement. They were told participation would not impact their grade in either direction, and students received no study incentive or extra credit. Furthermore, students were informed that all data would be anonymized, and they could withdraw their participation at any time.

### Setting and Sample

The setting was a COIL course in urban health at LDUC and community health at OSU, delivered to students asynchronously via the Canvas learning management system and synchronously via Zoom web-conferencing. The sample was undergraduate students at LDUC invited to attend focus group interviews after submitting the course and final grades. Fifteen out of 16 Norwegian students in their fourth semester participated at a rate of 93%. The focus group consisted of seven students in 2021 and eight students in 2022. Participants were female, with an age range of 23 to 31 (median = 26.4).

### Data Collection

Each focus group was interviewed once for 60 min over Zoom in June 2021 and June 2022. We developed a semi-structured interview guide based on pedagogical experience and knowledge gained from the Transformation Lab to facilitate focused discussion and allow prompts to expand participant comments and seek clarification of responses. A well-prepared academic moderator, not part of the research team, without student affiliations, but familiar with the field, conducted both focus groups to decrease the chance of biased responses and to ensure consistency (Morrison-Beedy et al., 2001). A technician familiar with COIL assisted with interview recordings.

### Data Analysis

The data analysis team consisted of researchers with pedagogy, instructional design, and nursing education backgrounds, all experienced in internationalization within higher education, enabling data interpretation from different academic standpoints. An example was the view on using icebreakers; the employee in the pedagogy specialization saw this as particularly important and a key factor for success.

Interviews were transcribed verbatim using voice-to-text software and then reviewed independently by three research team members, two nurse researchers, and one pedagogical expert to ensure the accuracy of the transcription and to uncover significant responses. Once verified, the team met to identify meaningful transcription responses. Out of a total of 234 relevant responses for the study aim, 180 responses were selected with interrater agreement greater than 95%. We then employed conventional content analysis approaches (Hsieh & Shannon, 2005), including compiling selected quotes into one data set and extracting each quote into common themes for within-group analysis. This action process was conducted after each focus group. After collecting the second focus group data, we conducted between-group analyses, including all data, to identify differences and similarities across the two cohorts.

## Findings

We identified two themes during the data analysis: “Virtual conversation builds collaborations and enhances learning” and “This opened my eyes.”

### Virtual Conversation Builds Collaborations and Enhances Learning

All students who participated in COIL in 2021 agreed on the importance of a synchronous first meeting as it gave a common platform and addressed the use of breakout rooms as an effective method to get to know each other. However, several students in 2022 commented on the absence of U.S. students at the initial meeting and pointed out that it was an unfortunate start. One student described this as making it difficult to get to know each other and meet later as they did not get to introduce themselves. Nevertheless, most in this cohort felt they could continue cooperating despite a difficult start.

Several students from both cohorts described the desire for synchronous information sessions so that they could understand each other’s academic arrangements. They also expressed the need for clear guidelines about which regular course components should be completed in the COIL project. One student stated: “the only thing we [in Norway] had to do was COIL. While they [the US students] had like clinical practice and other lectures outside the COIL project.” Another supported this, “I was thinking like they would do everything together with us.” LDUC’s students did not understand the U.S. education system. The consensus was the Norwegian students thought the U.S. students were in a similar program.

Several students reflected that they were doing the heavy lifting of communication by speaking in their non-native language and initially dreaded speaking English. Still, during the focus group, the majority said that the language difference was not a problem due to course organization. Icebreakers and breakout rooms were highlighted as helpful for easing students into conversing in a secondary language. Also, the small group size, less than four, contributed to overcoming language barriers and nervousness. One student appreciated the U.S. students: “I feel like the Americans were so nice and polite due to the language barrier. Like they were so patient, and it made me feel okay. . . . .”

Furthermore, icebreaking activities gave the students an opportunity to get to know each other and collaborate before the project started. As one student said: “*The icebreakers made a good start to get to know each other.*” However, some students had problems finding suitable times to connect with the U.S. students and pointed out that it would be advantageous for the course if a time schedule were determined in advance. Not meeting virtually contributed to not developing a relationship with their peers. The collaboration worked well for most groups; they met virtually outside the planned times noted in the syllabus. The students who took this approach indicated they became good friends with the U.S. students and gained an international network through COIL engagement.

Students carried out group chats throughout the COIL project and participated in synchronous and asynchronous meetings that served as a solid base for developing an effective and safe relationship. A few students needed help to make common times for the group work and stated more meetings should be mandatory for all who had been accepted into COIL to facilitate effective relationships with their international student colleagues.

Most students said the value of COIL extended beyond the classroom’s curricular requirements. Via their virtual conversations and videos made by U.S. students, Norwegian students learned about U.S. culture and how people live in the United States. One student said:

My favorite part was just getting to know people from another country and being able to collaborate with them. And it was fun doing the project, but I really liked all the conversations outside the project, like how they live, how their school system is, and how they are doing like nursing studies after bachelor . . . just getting to know them really and sharing pictures from everyday life and stuff.

The student groups were free to find their own way to collaborate. Most groups chose synchronous, virtual meetings that allowed them to meet and address different topics on homelessness, drug addiction, and the marginalization of people living in inner cities. Students agreed they learned a lot through the virtual discussions. They could verbally share knowledge, address and discuss problems, and compare health care systems. According to most of those interviewed, the overseas virtual discussions contributed to enhanced knowledge and understanding of culture and marginalized populations. For example, students became more aware of services offered to marginalized people in the US, saying, “For instance, the SNAP [supplemental nutrition assistance program] program that they have which is for nutritional food benefits for low-income families or those in need” and “things that they had that we didn’t have that we maybe should have.”

Another expressed the importance of getting information directly from someone who knows the U.S. health care system, “. . . [It is] interesting to see how the different countries are meeting the different issues and problems, so they had some differences in the way they were meeting [marginalized groups] that I also thought was some very good things.”

Extracurricular topics were also addressed,

I liked the discussions we had within our group when we had time to actually talk about things. For example, we discussed gun laws and how easy it is to get a weapon in the States and how difficult it is in Norway, which is not related to the subject.

### This Opened My Eyes

COIL helped the students gain a new understanding of the U.S. health care system and culture. They described the knowledge gained during the collaboration as an eye-opener that broadened their global perspectives. Acquisition of knowledge about different international approaches to marginalization through discussions and research articles gave new perspectives. As one student stated:

I have a lot of prejudices to the health care system in the US. Because ever since I was a small kid, you always like hear, like yeah, if you get cancer in the USA, you must pay millions and millions—people die because they cannot pay.

Many students had the same preconceived ideas. They noted that sharing knowledge globally with their U.S. counterparts contributed to a different view of the U.S. health care system, demonstrating the COIL experience led to cultural knowledge growth. The students also agreed that the discussions where they compared, discussed problems and solutions, and gave new perspectives expanded their learning. One student said, “We also get a better understanding that like each country is different. So well, it opened my eyes. . .” Students expressed that COIL exposed them to several valuable ideas to implement in Norway.

COIL provided Norwegian nursing students a unique opportunity to get real-life stories from their U.S. peers. Most students said that their information about the United States comes from films and news. None of the students had previously lived or worked in the United States. One student said, “We hear a lot of bad news about the US or the things that are happening in the US.” Furthermore, she noted that virtual conversations with the U.S. students contributed to new knowledge and perspectives and helped her recognize that more resources are aimed at marginalized citizens than previously thought. The real-life stories led to a deeper understanding of the U.S. health care system and culture.

The virtual meetings gave students an opportunity to communicate, ask questions, compare, and understand how the health care systems handle marginalized citizens in both countries. One said, “It was really good to see the difference between OSU and LDUC when it comes at least to our subject.” Another commented on all the various resources offered to people with substance use disorders in the United States, *“*We looked specifically on drug addicts, and I was quite impressed by everything, like by every offer that’s out there.” Direct communication with U.S. students gave Norwegian students an expanded lens of the health care system outside of Norway and recognition that they held some misconceptions prior to their interaction.

## Discussion

This study described the experience of Norwegian nursing students with internationalization through participation in a COIL course. Our main findings are that students benefited from the cultural encounters provided by COIL, learning about cultural differences and similarities and challenging prior assumptions, thereby increasing the desire for further international interactions in accordance with Campinha-Bacote, (2018).

The icebreakers and the synchronous, small breakout room sessions created a safe space, easing the language barrier and allowing students to build trusting relationships with each other. In line with our findings, previous studies have found that icebreakers could increase student motivation for learning, help students form relationships, and permit students to speak foreign languages without being concerned about vocabulary (Chowdhury, 2022; Kavanagh et al., 2011; Pratama et al., 2021). A safe learning environment where teachers and students work together is fundamental to communication, learning, developing collaborative skills, and collaboration to work (Mikkonen et al., 2015; Torres Martín et al., 2021). The use of icebreakers and synchronous break-out room sessions, the cultural encounters, opened an arena for cultural knowledge, skill, interaction, and desire to develop (Campinha-Bacote, 2018).

Some students also expressed a desire for more time to simply talk about whatever they wished outside of the course content. In line with Campinha-Bacote, (2020) theory about cultural desire, the students wanted to connect and learn about U.S. culture. In Spring 2021, students in Norway and the United States were stuck at home, feeling isolated, due to the pandemic, and thus, appreciated being able to connect to the world and have social contact through COIL. In contrast, in 2022, as the pandemic was resolved and people were venturing into their communities again, our participants were juggling the completion of clinical education at clinical sites along with didactic coursework, so COIL was not prioritized as it was before. Thus, we learned that data from COIL research must be contextualized to be fully understood.

COIL could unite students to collaborate and enhance global competence across international borders (Onoverole & Fowler, 2021). Our students expressed learning beyond COIL and gained further knowledge about U.S. culture and lifestyle, nursing education, and employment in the United States, contributing to cultural knowledge and skills. Informed by Campinha-Bacote (2020) theory, our findings suggest that virtual face-to-face cultural encounters engage students, making them want to learn more and creating cultural desire.

Students indicate their appreciation of learning from their U.S. peers who were cultural informants rather than previous knowledge and perceptions about the United States from the Norwegian media and other news sources. This conveys an increase in cultural knowledge gained through cultural encounters where students shared, discussed assumptions, and learned from each other. It also suggests that COIL contributed to cultural humility and the creation of a different view of culture and the health care system. Informed by Campinha-Bacote’s (2020) theory, our findings suggest that knowledge acquisition and new perspectives regarding different international approaches to marginalization were gained through COIL via planned discussions, research articles, and presentations, leading to emerging cultural awareness.

Mandatory meeting dates for students are essential (Bingen et al., 2019). Some of our students noted there were challenges in attending scheduled meeting times that could impede the group in forming a relationship and a productive partnership for learning. This might decrease cultural learning, cultural skills, and cultural desire. On the other hand, other students planned virtual social meetings outside of scheduled COIL times, demonstrating their cultural desire leading to forming new international friendships. This shows that COIL can be a valuable teaching/learning method that brings cultural knowledge and skills to students.

Our findings suggest that COIL is a learning method that could prepare nursing students to deliver culturally congruent health care by facilitating cultural awareness, humility, knowledge, skill, and desire. Consequently, through cultural encounters students could experience a new way of thinking about preconceived ideas developed.

### Lessons Learned and Recommended Best Practices

Developing similarity in course syllabi in both countries enhances collaboration and makes it easy for students to choose a topic for group work and to compare the different perspectives. Choosing courses with similar learning content is recommended so that students will reach the learning goals of their respective universities (Jansen, 2015).

To support student commitment, faculty could help students prioritize COIL by having a clear framework, such as dates for pre-COIL, synchronous and asynchronous meetings, and clarifying COIL expectations. They should ensure that COIL meeting days/times will be reserved only for COIL activities, whereby all students and faculty are virtual and not at a clinical site. These provisions in both countries could increase time and attention for students to connect and work together on the COIL project during a scheduled time. As per Bingen et al. (2019), our study underlines the importance of faculty support and a predictable structure that facilitates learning.

Students were placed in groups early to get to know each other before starting the project. However, in 2022, some students requested group changes due to scheduling issues, leading to a lack of rapport and group cohesiveness since group dynamics changed. Some students pointed out that more mandatory synchronous meetings would be helpful for collaboration, which suggests that students needed help and social assistance. Consequently, faculty will develop a best practice communication and group work checklist as a resource for students. Assigned groups will also be expected to stay consistent throughout to promote teamwork, reduce confusion, and support students in staying on track with the COIL project. Despite identical learning content, some students faced challenges in the execution of the COIL project due to varying requirements from their respective universities, as well as employment and family obligations. Therefore, we believe that clarifying COIL expectations to students in the application process, such as scheduled COIL meetings and the need to be flexible in meeting outside of scheduled COIL time, could be a proactive strategy in setting the groundwork for working collaboratively on the COIL Urban Health project.

In meeting demands outside COIL, such as other classes, family, and work obligations, students had to overcome a six-hour time zone difference and find suitable times to collaborate. The time difference was referred to in interviews from both years and is a known challenge in virtual exchange projects across time zones (Misra et al., 2020). To overcome the time difference challenge, giving students flexibility in their schedules is essential to plan for collaboration across time zones. On the other hand, different time zones can contribute to the students becoming more creative, finding asynchronous collaboration methods, for instance, through the common student learning management system Canvas. Thus, balancing time zone challenges with flexibility and creativity is necessary to obtain optimum student outcomes.

### Limitations

The first focus groups occurred early in the COVID-19 pandemic when students were restricted to at-home learning. Students were eager to engage with others and had considerable flexibility in scheduling. The second focus group occurred when the students re-entered face-to-face educational and clinical experiences. This transition meant they were trying to balance many adjustments returning to pre-COVID-19 education, clinical, and social interactions; furthermore, some had challenges finding adequate time to engage in COIL and focus group activities. While 15 of 16 COIL students participated in the focus group, their interactions and responses may not reflect those of a larger, more diverse sample of students who did not apply to be part of the experience. The sample was limited to 15 female undergraduate students who were in their fourth semester. Their responses may not reflect male nursing students or students at varying levels of study. However, the students who participated in COIL shared positive and negative experiences. The sample was considered to generate sufficient information power for qualitative research (Malterud et al., 2016).

## Conclusion

This study described the experience of Norwegian nursing students with internationalization through participation in a COIL course. First, our data supported the theme that *“virtual conversation builds collaborations and enhances learning.”* Students enjoyed connecting with peers outside their own country and felt these conversations built a communication pathway that facilitated collaboration and true partnership, indicating that their cultural encounters led to cultural desire. The ability for students to engage and learn from each other and build knowledge based on real-life experiences rather than from movies or social media was evident and valued. Overall, the Norwegian students who participated in a U.S.–Norway COIL project acknowledged they had learned transferable lessons from their global partners that could be applied to the patient care of the homeless population in Norway. COIL projects offer unique and logistically feasible ways to connect students globally to broaden their understanding of best practices and shared contributions to improving patient outcomes. Second, students held preconceived ideas about the U.S. health care system and culture, and these misconceptions were challenged and clarified in these COIL experiences. These COIL experiences “*opened their eyes*” to a new way of thinking about preconceived ideas developed without true knowledge of situations outside their country, demonstrating these cultural encounters led to cultural awareness, humility, knowledge, skill, and desire.

### Future Research

Future research and educational endeavors could include COIL projects embedded across additional coursework and curricula at the undergraduate and graduate levels. An important next step in development would be to include more students, moving beyond students who volunteer to participate in COIL projects to requiring COIL in coursework components with careful faculty support to match the challenge. Adding quantitative approaches to qualitative ones would enhance data collection and interpretation robustness. Future steps also include investigation of impact on faculty teams who also learn and grow together through preparation and leading COIL courses together. Furthermore, we intend to develop future COIL courses as a flexible, cost-effective, and environmentally friendly option to study abroad.
